# Relationships Between the Usage of Televisions, Computers, and Mobile Phones and the Quality of Sleep in a Chinese Population: Community-Based Cross-Sectional Study

**DOI:** 10.2196/18095

**Published:** 2020-07-07

**Authors:** Yao Jie Xie, Daphne SK Cheung, Alice Y Loke, Bernice L Nogueira, Karry M Liu, Angela YM Leung, Alice SM Tsang, Cindy SU Leong, Alex Molassiotis

**Affiliations:** 1 School of Nursing The Hong Kong Polytechnic University Hong Kong China (Hong Kong); 2 Centre for Gerontological Nursing, School of Nursing The Hong Kong Polytechnic University Hong Kong China (Hong Kong); 3 School of Health Sciences and Sports Macao Polytechnic Institute Macau Macao; 4 World Health Organization Collaborating Centre for Community Health Services, School of Nursing The Hong Kong Polytechnic University Hong Kong China (Hong Kong); 5 Kiang Wu Nursing College of Macau Macau Macao

**Keywords:** electronic device, screen-based, sleep, Chinese, digital, mobile phone

## Abstract

**Background:**

No study has comprehensively investigated the association between the usage of typical screen-based electronic media devices and sleep quality in a Chinese population with individuals in a wide range of ages.

**Objective:**

This study aimed to understand the characteristics of television (TV) viewing, computer usage, and mobile phone usage in a representative Chinese population in Macau and to examine their roles in predicting the variations in sleep quality.

**Methods:**

This cross-sectional study was an analysis of 1500 Macau residents aged 15 to 90 years based on a community-based health needs assessment study entitled, “Healthy Living, Longer Lives.” Data collection was conducted in 7 districts of Macau from 2017 to 2018 through face-to-face interviews. The durations of daily TV viewing, computer usage, and mobile phone usage were recorded in a self-administered questionnaire. The Chinese version of the Pittsburgh Sleep Quality Index (PSQI) was used to assess the sleep quality.

**Results:**

The prevalence of TV, computer, and mobile phone usage was 78.4% (1176/1500), 51.6% (769/1490), and 85.5% (1276/1492), respectively. The average daily hours of usage were 1.75 (1.62), 1.53 (2.26), and 2.85 (2.47) hours, respectively. Females spent more time watching TV (*P*=.03) and using mobile phones (*P*=.02) and less time on the computer (*P*=.04) as compared to males. Older adults were more likely to watch TV while young people spent more time using the computer and mobile phones (*P* for all trends<.001). The mean PSQI global score was 4.79 (2.80) among the participants. Females exhibited significantly higher PSQI scores than males (5.04 vs 4.49, respectively; *P*<.001). No linear association was observed between the PSQI score and the amount of time spent on the 3 electronic devices (*P*=.58 for PSQI-TV, *P*=.05 for PSQI-computer, and *P*=.52 for PSQI-mobile phone). Curve estimation showed significant quadratic curvilinear associations in PSQI-TV (*P*=.003) and PSQI-computer (*P*<.001) among all the participants and in PSQI-mobile phone among youths (age, 15-24 years; *P*=.04). After adjustment of the gender, age, body mass index, demographics, and lifestyle factors, more than 3 hours of TV viewing and 4 hours of computer usage or mobile phone usage was associated with 85% (95% CI 1.04-1.87; *P*=.008), 72% (95% CI 1.01-2.92; *P*=.045), and 53% (95% CI 1.06-2.22; *P*=.03) greater odds of having poor sleep quality (PSQI score>5), respectively.

**Conclusions:**

The mobile phone was the most popular screen-based electronic device used in the Macau population, especially among young people. “J” shape associations were observed between sleep quality and the duration of TV viewing, computer usage, and mobile phone usage, indicating that the extreme use of screen-based electronic devices predicted poorer sleep status, whereas moderate use would be acceptable.

## Introduction

The use of screen-based electronic media devices such as computers, televisions (TVs), mobile phones, video games, and other screen-based products have become an integral part of our lives and has continued to increase sharply in the past decade [1,2]. For example, about 80% of the population use smartphones and about 90% of the population watch TV in the United States of America, the United Kingdom, Mainland China, and Hong Kong [2]. However, the pervasive use of screen-based electronic media devices is a likely contributor to widespread sleep problems [3].

Numerous studies across the countries have suggested that prolonged daily use of screen-based electronic devices lead to negative sleep outcomes, including decreased sleep duration, reduced sleep efficiency, increased daytime sleepiness, and sleep disturbances [1,4,5]. A recent systematic review showed that in over 90% of the studies, increased screen-based media consumption by children and adolescents (age, 5-17 years) was associated with a reduction in total sleep duration [3]. Another systematic review of 67 studies in school-aged youth and teenagers found that screen time was associated adversely with sleep health through delayed bedtimes and reduced sleep duration [6]. Regarding the Chinese population, a cross-sectional study on adolescents in Hong Kong showed that prolonged mobile phone use was significantly related to daytime sleepiness, decreased sleep duration, and poor sleep quality [7]. Similar sleep problems were reported among the residents of Macau, which is another special administrative region of China. Nearly 40% of the adults in Macau reported at least one type of sleep disturbance [8]. We believe that most residents of Macau use electronic media devices because the cultural customs and lifestyle of Macau are similar to those of Hong Kong. However, no study has examined the role of screen-based electronic device usage in the development of sleep problems in Macau.

The majority of the previous studies focused on a special age group—in particular, the age groups of children and adolescents [1,3,9-12]. Very few studies have targeted a population of a wide age range. Youths are not the only age group that use screen-based electronic devices. The different pattern of use in adulthood may reflect a different sleep health profile. For example, adults may seek compensatory sleep when spending too much time on screen-based media devices, and thus, media use is associated with sleep onset latency but not tiredness [13]. Many of the previous studies focused only on 1 or 2 screen-based electronic devices [3,10,14,15]. It is also not known whether the same pattern of sleep problems occurs with the usage of different screen-based electronic media devices [1]. In fact, the trend in the modern society is the simultaneous use of multiple screen-based electronic devices and thus, a comprehensive investigation of several screen-based electronic media devices may help us to better understand the overall situation [6].

We conducted this study with the following aims: (1) describe the usage of 3 major screen-based electronic media devices (TV, computer, and mobile phone) and the sleep quality in a representative Chinese population in Macau, (2) examine the patterns of the associations between electronic device usage and sleep quality, and (3) explore how the usage of electronic devices predicts poor sleep status.

## Methods

### Data Source and Study Design

This study is a report of the partial data from a project named “Healthy life, longer lives (HLLL),” which was conducted from 2017 to 2018 in Macau. HLLL is a health needs assessment study of the Macau civilian, noninstitutionalized population. It was designed to assess the lifestyle behaviors of Macau residents in terms of their dietary habits, sleep patterns, physical activity, electronic device usage, smoking habits, drinking habits, and health information seeking behaviors, and to identify the barriers for a healthy lifestyle in vulnerable groups. The ethical approval for the study was obtained from the Human Research Committee of Hong Kong Polytechnic University (HSEARS20180516001), the Macau Polytechnic Institution (07/PSC/ESS/2017), and the Kiang Wu Nursing College of Macau (REC–2017.3). All participants signed informed consent forms.

### Participants and Sampling Method

The inclusion criteria of the participants were (1) aged 15 years or above, (2) can read and write Chinese and speak Cantonese or Putonghua, which are the dialects commonly used in the city, and (3) residing in one of the 7 parishes in Macau. The exclusion criteria were (1) nonpermanent residents, (2) unable to communicate owing to psychological or physical illness, and (3) cannot provide written informed consent.

A multiple-stage sampling frame according to gender and age was adopted to recruit the participants. First, the number of the needed participants in each age group (age [years]: 15-17, 18-24, 25-34, 35-44, 45-54, 55-64, and 65+) was determined by referring to the distribution of age and gender in the census data of the Macau population [16]. Second, several recruitment strategies were adopted to approach potential participants in each age group. Thirty baccalaureate nursing students from 2 tertiary education institutions in Macau and living in different parishes of Macau were trained for recruitment and data collection. They went to their living districts and the nearby housing districts to recruit participants. They were also asked to invite 1-3 friends or relatives that belonged to the same age groups but with different education levels to participate in the study. To recruit enough adolescents (age, 15-17 years) and older adults (aged 65 years or above), the trained students also went to the public-funded health care and social service centers for adolescents and older adults for recruitment. To ensure the representativeness of the sample, only 1 eligible person in 1 household was considered as a valid participant. To avoid duplication, we asked the participants to provide the last 3 numbers of their Macau national identification numbers. These Macau identification numbers were stored in a separate data set and the numbers were checked thoroughly, and we did not find any duplicate cases. Upon successful contact with the target person and after obtaining written informed consent, the self-administered questionnaire was given to the participants to complete. If he/she could not read the questionnaire (eg, older adults), the student assistants conducted a face-to-face interview thereafter. According to the feedback from the student assistants, only a small number of older persons aged 75 years and above completed the questionnaires by interview, accounting to less than 1% of the whole sample.

### Measures

#### Screen-Based Electronic Media Device Usage

The screen-based electronic devices analyzed in this study were the TV, computer, and mobile phone, as these are the 3 most frequently used screen-based electronic media devices. Respondents were asked how many days they spent watching TV or using a computer and mobile phone in 1 week and the duration they spent on TV viewing and computer and mobile phone usage on a typical day (minutes or hours/day). Minutes were converted to hours and recorded to the nearest 0.1 hours. The upper bounds of the duration of each electronic device usage was set as 16 hours, because we considered that one person might spend 8 hours for sleep and for other things in a day. The total number of hours spent on the 3 electronic devices in 1 day should not be beyond 24 hours. The student assistants corrected the answers with the participants after receiving the questionnaires to avoid any unreasonable value. The average time reported for using each electronic device every day was calculated by dividing the sum of the hours spent on these devices in 1 week divided by 7 (ie, 7 days in a week).

#### Sleep Quality

The Chinese version of the Pittsburgh Sleep Quality Index (PSQI) [17,18] was used to measure the sleep quality. The PSQI has 7 components, namely, (1) sleep duration, (2) sleep disturbance, (3) sleep latency, (4) daytime dysfunction due to sleepiness, (5) sleep efficiency, (6) overall sleep quality, and (7) sleep medication. The scale consists of 19 items, and each item is scored from 0 to 3. The PSQI global score was calculated from the 7 component scores. The score ranges from 0 to 21. Higher PSQI global score indicates poorer subjective sleep quality. The Chinese version of PSQI has been identified with good reliability (*r*=0.82-0.83) [17]. A global PSQI score >5 is considered as poor sleep quality with a sensitivity of 98% and a specificity of 55% [17], indicating good performance when the PSQI is used in the Chinese population.

#### Demographics and Anthropometric Measures

Information on the following demographic characteristics were solicited: gender (male or female), age (years), and age groups (age [years]: 15-17, 18-24, 25-34, 35-44, 45-54, 55-64, and 65+), living region (7 parishes of Macau), employment status (ie, employed, retired, student, housewife, unable to work, unemployed), marital status (ie, never married, married, divorced, widowed, others), education level (ie, primary or below, secondary, tertiary or above), and monthly household income (in MOP, conversion rate: 1MOP=US $0.125). Participants reported their weights and heights by kilogram and centimeters, respectively. Body mass index (BMI) was calculated as weight in kilograms divided by the square of height in meters. Participants were categorized as underweight (BMI<18.5 kg/m^2^), normal weight (18.5 kg/m^2^≤BMI<23.0 kg/m^2^), overweight (23.0 kg/m^2^≤BMI<25.0 kg/m^2^), and obese (BMI≥25.0 kg/m^2^) according to the World Health Organization (WHO) standard for Asian populations [19]. The former 3 categories were designated as nonobese (BMI<25.0 kg/m^2^) in some analyses to compare with the obese group (BMI≥25.0 kg/m^2^).

#### Other Covariates

The following lifestyle factors were considered as potential covariates and measured in the questionnaire: physical activity (insufficient/sufficient according to WHO standards), smoking (currently smoking or not), drinking (currently drinking or not), and unhealthy eating habits in terms of remaining hungry (eg, delaying or skipping meal), dieting (eg, weight losing, fasting), and overeating. The eating habits were measured using the 5-point Likert scale ranging from Never (1), Occasionally (2), Sometimes (3), Frequently (4), to Always (5).

### Statistical Analysis

The basic characteristics of the participants were described by the mean and SD for the continuous variables and by count and percentage for the categorical variables. To describe the usage of the device and the sleep quality, the duration of usage (hours), status of usage (yes/no), global score of the PSQI, and poor sleep status were presented by mean (SD) or frequency (n [%]) where appropriate. The device usage and sleep quality according to the different demographical groups were analyzed using one-way analysis of variance (ANOVA) or independent *t*-test for the continuous variables and chi-square test for the categorical variables. The mean sleep duration (hours) and the average 7 component scores of the PSQI were presented and compared with gender and age groups by ANOVA. To examine the association between screen-based electronic device usage and sleep quality, the average number of hours spent in using electronic devices were categorized into different strata and ANOVA was used to compare the differences in the PSQI scores among the groups. Both linear and curve estimation models were performed to demonstrate the nature of the association between using electronic devices and sleep quality. Binary logistic regression analysis was adopted to determine the predictive role of the electronic device usage on poor sleep status. The dependent variable was poor sleep quality (PSQI score>5). Odds ratios (ORs) and 95% CIs for having poor sleep quality according to different durations of using the computer, mobile phone, and TV were calculated accordingly. SPSS 25.0 software (IBM Corp) was used in all data analyses and the significance level was considered as .05.

## Results

### General Characteristics of the Participants

A total of 1500 participants were recruited between 2017 and 2018. The mean age of the females was 39.1 years and that of males was 39.3 years. The 4 age groups were as follows: youth (15-24 years), young adults (25-44 years), middle-aged adults (45-64 years), and older adults (65 or above). The proportions of the 4 age groups were as follows: youth, 14.1% (212/1500); young adults, 35.4% (531/1500); middle-aged adults, 37.4% (561/1500); and older adults, 13.1% (196/1500). The majority of the participants were employed (1030/1473, 70.0%), had secondary or above education level (1206/1489, 81.0%), and were married or they had a partner (899/1487, 60.5%). Females had lower monthly household income than males (*P*<.001). Males were more likely to be overweight or obese than females (289/667, 43.3% vs 269/805, 33.4% respectively; *P*<.001). The mean BMIs of the males and females were 22.8 kg/m^2^ and 22.0 kg/m^2^, respectively (*P*<.001). Most participants did not have drinking and smoking habits, but the number of male drinkers was higher than that of female drinkers (221/675, 32.7% vs 152/821, 18.5%, respectively; *P*<.001). The number of male smokers was higher than that of the female smokers (97/673, 14.4% vs 23/821, 2.8%, respectively; *P*<.001). Around half of the male population (332/669, 49.6%) had enough physical activity while only 35.7% (286/801) of the female population was involved in any physical activity. Over half of the participants reported that they had at least one unhealthy eating habit, and males showed a higher percentage of unhealthy eating habits than females (401/669, 59.9% vs 420/819, 51.3%, respectively; *P*<.001). The basic characteristics of the participants according to gender are shown in Table 1.

**Table 1:** Characteristics of the participants (N=1500).

Characteristics		Males	Females
Age (years), mean (SD)		39.3 (17.5)	39.1 (16.8)
Length of stay in Macau (years), mean (SD)^a^		36.4 (15.8)	33.1 (15.2)
BMI, mean (SD)^a^		22.8 (3.1)	22.0 (3.3)
**Age group (years), n (%)**			
	Youth (15-24)	102 (15.1)	110 (13.3)
	Young adults (25-44)	240 (35.5)	291 (35.3)
	Middle-aged adults (45-64)	241 (35.7)	320 (38.8)
	Older adults (≥65)	93 (13.8)	103 (12.5)
**Marital status, n (%)**			
	Single/Divorced/Separated	261 (38.7)	327 (40.3)
	Married/with partner	414 (61.3)	485 (59.7)
**Education level, n (%)**			
	Primary or lower	125 (18.5)	158 (19.4)
	Secondary	334 (49.6)	428 (52.5)
	Tertiary or higher	215 (31.9)	229 (28.1)
**Employment status, n (%)^a^**			
	Employed	468 (70.5)	562 (69.5)
	Retired	82 (12.3)	90 (11.1)
	Student	73 (11.0)	69 (8.5)
	Others (housewife, unable to work, unemployed)	41 (6.2)	88 (10.9)
**Monthly household income (MOP)^b^, n (%)^c^**			
	≤MOP $9999	26 (3.9)	83 (10.2)
	MOP $10,000-MOP $29,999	275 (40.9)	349 (42.8)
	MOP $30,000-MOP $59,999	196 (29.2)	217 (26.6)
	≥MOP $60,000	66 (9.8)	49 (6.0)
	Unknown	109 (16.2)	117 (14.4)
**BMI, n (%)^c^**			
	Underweight (≤18.5 kg/m^2^)	38 (5.7)	100 (12.4)
	Normal (18.6-22.9 kg/m^2^)	340 (51.0)	436 (54.2)
	Overweight (23-24.9 kg/m^2^)	159 (23.8)	132 (16.4)
	Obese (≥25 kg/m^2^)	130 (19.5)	137(17.0)
**Lifestyle behaviors, n (%)^c^**			
	Sufficient physical activity^d^	332 (49.6)	286 (35.7)
	Unhealthy eating habits^e^	401 (59.9)	420 (51.3)
	Drinking	221 (32.7)	152 (18.5)
	Smoking	97 (14.4)	23 (2.8)

^a^*P*<.001.

^b^MOP: Macau Pataca; 1MOP = US $0.125.

^c^*P*<.001.

^d^According to WHO (2018), sufficient physical activity means doing at least 150 minutes of moderate-intensity aerobic physical activity or at least 75 minutes of vigorous-intensity aerobic physical activity throughout the week; *P*=.001.

^e^Unhealthy eating habit means remaining hungry (eg, delaying or skipping meal), dieting (eg, weight losing, fasting), or overeating.

### Screen-Based Electronic Media Device Usage Among Participants

Among the 1500 participants, the prevalence of watching TV and using computer and mobile phones was 78.4% (1176/1500), 51.6% (769/1490), and 85.5% (1276/1492), respectively. Multimedia Appendix 1 shows the duration of screen-based electronic device usage and sleep quality according to the demographics and lifestyle factors of the participants. The average number of daily hours of watching TV, using a computer, and using a mobile phone was 1.75 (1.62) hours, 1.53 (2.26) hours, and 2.85 (2.47) hours, respectively. Females spent more time watching TV (*P*=.03) and using mobile phones (*P*=.02) and less time on computers (*P*=.04) than men. The use of the 3 electronic devices showed trends according to age, with older adults being more likely to watch TV and young people spending more time using computers and mobile phones (*P* for all trends<.001). The youth spent the longest time on computers and mobile phones at 2.7 hours and 4.4 hours, respectively. Participants who were couples, had lower educational level, were retired or unemployed, and had lower monthly household income had longer TV viewing times, whereas participants who were single, had a higher education level, who were students, and who were employed reported longer computer and mobile phone usage (*P*<.001). Significant differences were also observed across the different BMI groups. Obese people had longer TV viewing time (*P*=.04) but shorter computer usage (*P*=.008) and mobile phone usage (*P*<.001) as compared to the nonobese people. Regarding the common lifestyle factors, participants with enough physical activity (*P*=.01) and drinking habits (*P*=.003) spent shorter durations on TV viewing, while those who had unhealthy eating habits spent a longer time on TV viewing (*P*<.001) and shorter time on computer (*P*=.001) and mobile phone (*P*<.001) usage. The number and percentage of the users of the 3 electronic devices according to demographics and lifestyle factors is shown in Multimedia Appendix 2.

### Sleep Quality of the Participants

The mean (SD) of the PSQI global score was 4.79 (2.80). Females had a significantly higher PSQI global score than males (5.04 vs 4.49; respectively; *P*<.001). The prevalence of poor sleeping quality in females was around 10% higher than that in males (308/794, 38.8% vs 181/642, 28.2%, respectively; *P*<.001). No statistically significant differences in the PSQI global scores were detected according to the age groups (*P*=.55), marital status (*P*=.22), education level (*P*=.07), and employment status (*P*=.06). The highest average PSQI global score was found in obese participants (5.36 (2.96); *P*<.001); 40.4% had poor sleep quality (*P*=.009). Those with unhealthy eating habits (*P*<.001), that is, remaining hungry, dieting, or overeating and smokers (*P*=.002) were more likely to have higher PSQI global scores. However, participants with different physical activity levels (*P*=.97) and drinking habits (*P*=.72) showed no significant difference in sleep quality (Multimedia Appendix 1). The sleep duration (hours) and the 7 component scores of the PSQI by gender and age groups are shown in Multimedia Appendix 3. Males had better subjective sleep quality (*P*=.007) and lower sleep disturbances (*P*=.01) and sleep latency (*P*<.001) than females. Youths had higher daytime dysfunction (*P*=.01) and sleep latency (*P*=.04) but lower sleep disturbances (*P*=.004) than older adults.

### Associations Between Screen-Based Electronic Device Usage and PSQI Scores

The associations between electronic device usage and sleep quality were examined by first comparing the mean global scores of PSQI with the stratified duration of electronic device usage. The results are shown in Table 2. Significant differences were found among TV viewing strata and computer usage strata. Those with 1.5-2.5 hours of viewing (PSQI: 4.37; *P*<.001) and 2.0-2.4 hours computer usage (PSQI: 4.25; *P*=.009) had the lowest PSQI scores. However, no significant difference was observed between PSQI scores among the different age groups of mobile phone users (*P*=.23). No linear association was found between sleep quality and the duration of usage of all 3 electronic devices (*P*=.58 for PSQI-TV, *P*=.05 for PSQI-computer, and *P*=.52 for PSQI-mobile phone). Thus, we conducted the curve estimation and observed significant quadratic curvilinear associations of sleep quality with the durations of using TV (*P*=.003) and computer (*P*<.001) but not the mobile phone (*P*=.12). We further analyzed the association in each age group for the mobile phone usage and found a significant quadratic association between mobile phone use time and sleep quality among youths (*P*=.04). We further examined the model fitting through the scatterplot of residuals by fit values for the linear model and quadratic model, respectively. We found that these linear models would be inadequate because their coefficients did not make practical sense, and residuals were not independent of the fit values. The quadratic models did not have these problems, and hence we considered the quadratic models to be the more reasonable models that appropriately described the associations of sleep quality with TV viewing, computer usage, and mobile phone usage. The quadratic associations could be demonstrated using the following equations:

PSQI global score = 5.033 – 0.268*duration of TV viewing + 0.040*duration of TV viewing²

PSQI global score = 5.044 – 0.336*duration of computer usage + 0.036*duration of computer usage²

PSQI global score = 4.644 – 0.013*duration of smartphone usage + 0.011*duration of smartphone usage² (age group 15-24 years)

**Figure 1:**
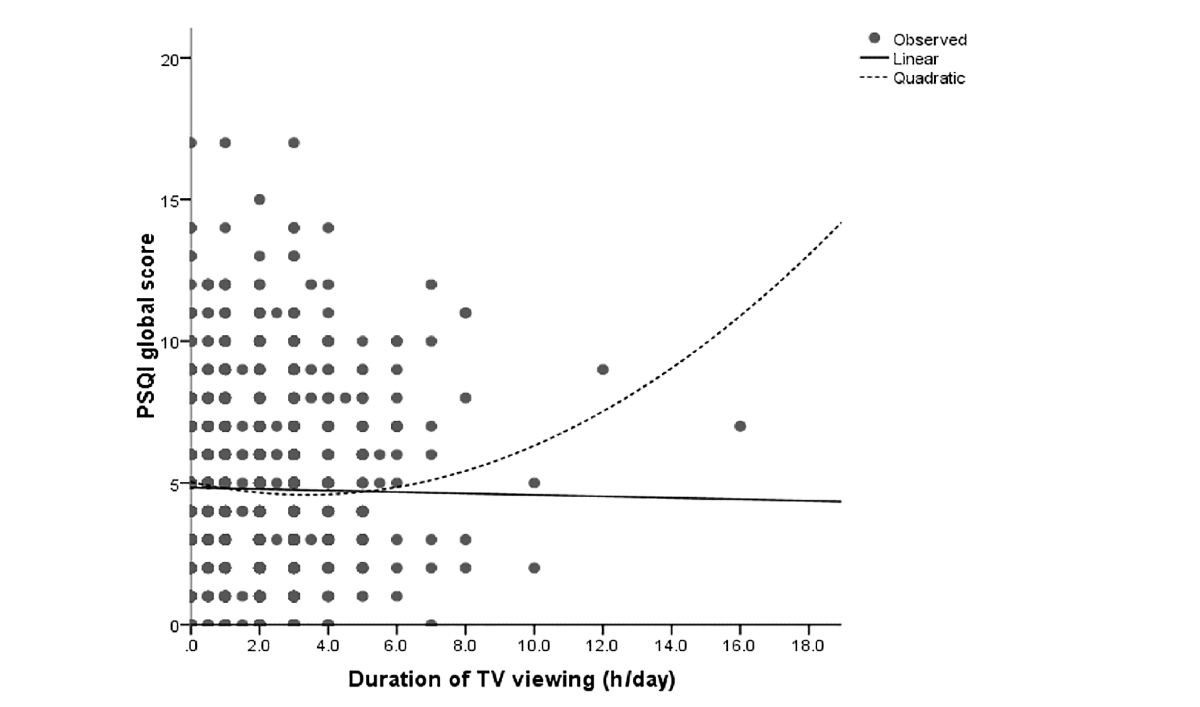
Curve estimation of duration of TV viewing with PSQI global score. PSQI: Pittsburgh Sleep Quality Index; TV: television.

**Figure 2:**
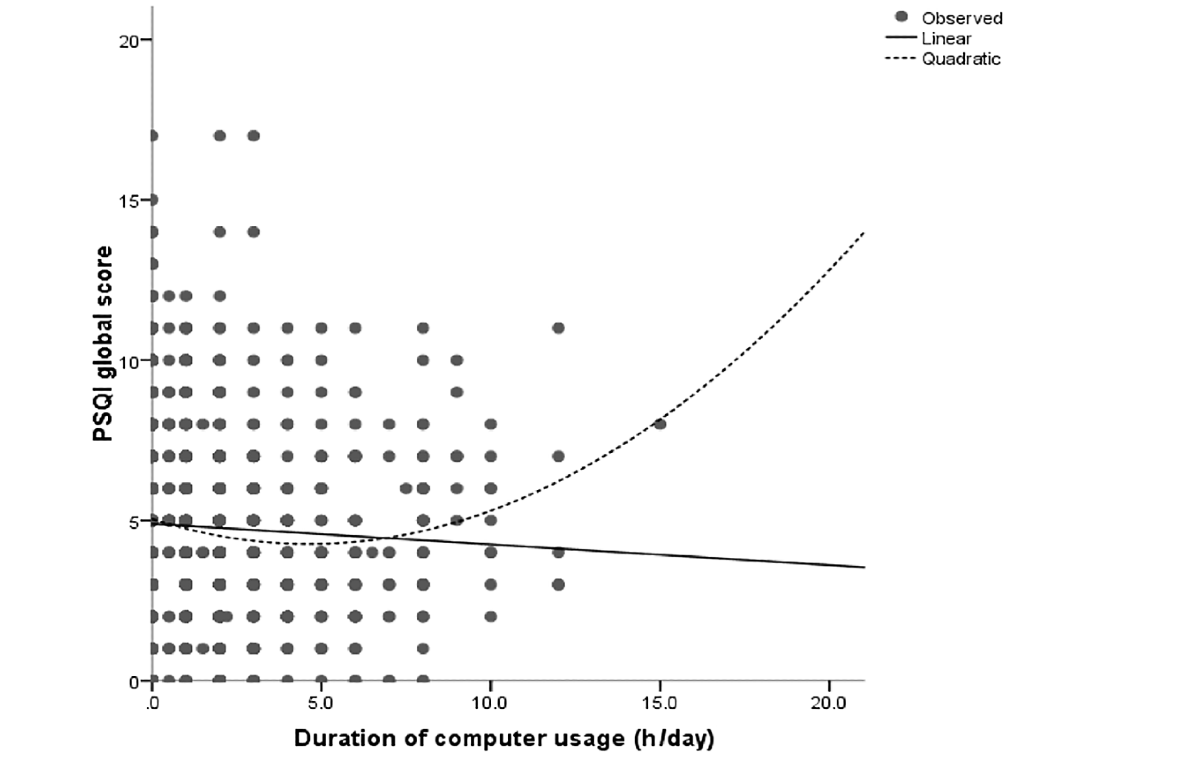
Curve estimation of duration of computer usage with PSQI global score. PSQI: Pittsburgh Sleep Quality Index.

**Figure 3:**
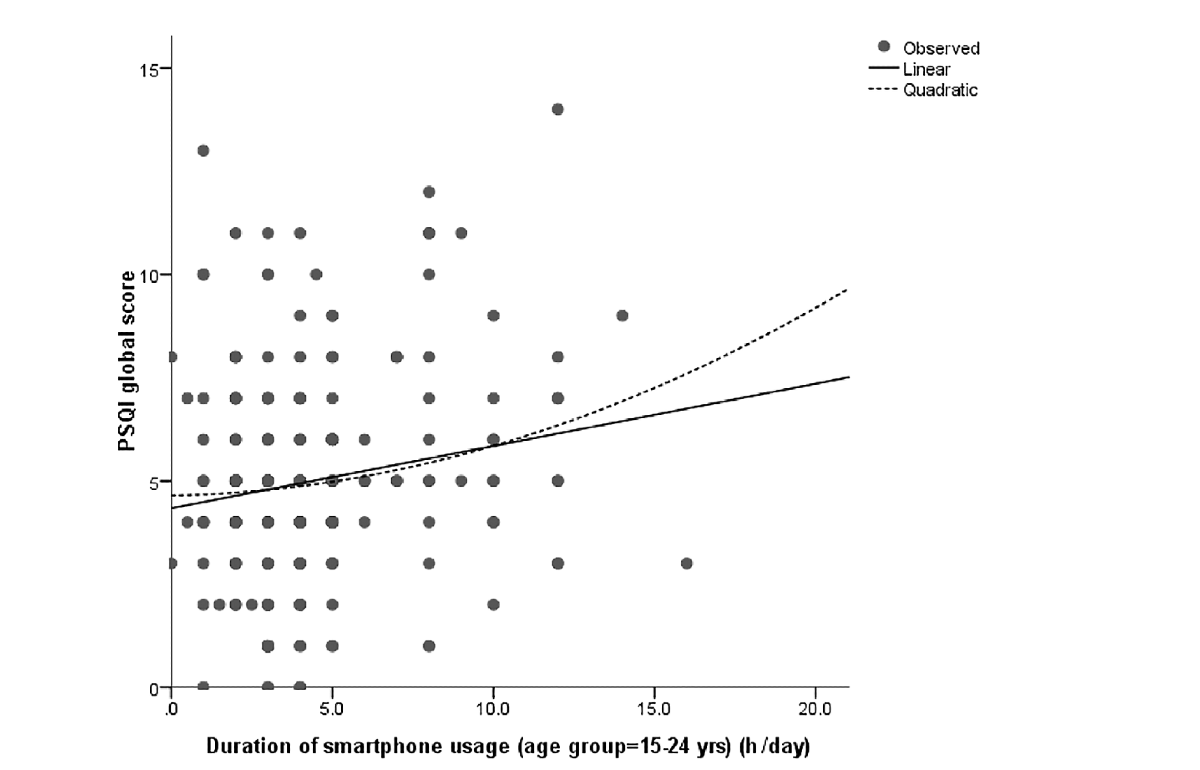
Curve estimation of the duration of mobile phone usage with PSQI global score for the age group of 15-24 years. PSQI: Pittsburgh Sleep Quality Index.

The association looks like a “J” shape. Both the shorter and longer use of TV, computer, and mobile phone were associated with increased PSQI scores (Figure 1-3).

**Table 2:** Univariate analysis of the duration of electronic media device usage with sleep quality (PSQI^a^ global score).

Duration of device usage			Total (n)		PSQI global scores, mean (SD)		*P* value
**Television^b^**						<.001	
	Nonuser	308		5.05 (2.88)			
	<1.5 h	394		4.91 (2.79)			
	1.5-2.5 h	345		4.37 (2.53)			
	2.5-3 h	213		4.42 (2.96)			
	>3 h	160		5.13 (2.89)			
**Computer^c^**						.009	
	Nonuser	685		5.02 (2.91)			
	<2 h	427		4.70 (2.69)			
	2-4 h	164		4.25 (2.66)			
	>4 h	143		4.64 (2.60)			
**Mobile phone^d^**						.27	
	Nonuser	202		4.88 (2.82)			
	<2 h	552		4.81 (2.74)			
	2-4 h	365		4.57 (2.80)			
	>4 h	305		4.98 (2.87)			

^a^PSQI: Pittsburgh Sleep Quality Index.

^b^No linear association; *P*=.58; quadratic: *P*=.003.

^c^No linear association; *P*=.05; quadratic: *P*<.001.

^d^No linear association; *P*=.52; not quadratic: *P*=.12.

### Role of Screen-Based Electronic Device Usage in Predicting Poor Sleep Quality

According to the stratified analyses and curve estimations, the lowest PSQI score occurred in the participants with a moderate duration of using electronic devices. We thereby used 1.5-3 hours of TV viewing and 2-4 hours of computer and mobile phone usage as the reference groups. We conducted binary logistic regression for analyzing the association of poor sleep status with electronic device usage. As shown in Table 3, except for those using mobile phones less than 2 hours, all other groups showed statistically significant associations. The crude ORs ranged from 1.43 to 1.78. After adjustment for gender, age, BMI, demographics, and lifestyle factors, the strength of the associations was slightly changed but they still showed statistical significance. The OR of poor sleep quality was 38% (95% CI 1.03-1.84; *P*=.03) and 85% (95% CI 1.18-2.91; *P*=.008) in the <1.5-hours and >3-hours TV viewing groups, respectively. In the computer usage groups, the OR was 71% (95% CI 1.10-2.64; *P*=.02) higher for <2-hours group and 72% (95% CI 1.01-2.92; *P*=.04) higher for the >4-hours group. As for the mobile phone usage, the adjusted OR was 1.53 (95% CI 1.06-2.22; *P*=.02) for the group with daily mobile phone use longer than 4 hours.

**Table 3:** Multiple logistic regression models for testing the association between the duration of daily electronic media device usage and poor sleep quality.

		Model 1^a^			Model 2^b^			Model 3^c^	
Device, Usage group		OR^d^ (95% CI)	*P* value	OR (95% CI)		*P* value	OR (95% CI)		*P* value
**Television**									
	1.5-3 h	1		1			1		
	<1.5 h	1.55 (1.22-1.97)	<.001	1.50 (1.15-1.94)		.003	1.38 (1.03-1.84)		.03
	>3 h	1.78 (1.23-2.57)	.002	1.89 (1.29-2.77)		.001	1.85 (1.18-2.91)		.008
**Computer**									
	2-4 h	1		1			1		
	<2 h	1.63 (1.12-2.37)	.01	1.66 (1.12-2.46)		.01	1.71 (1.10-2.64)		.02
	>4 h	1.66 (1.02-2.72)	.04	1.74 (1.03-2.93)		.04	1.72 (1.01–2.92)		.04
**Mobile phone**									
	2-4 h	1		1			1		
	<2 h	1.13 (0.86-1.47)	.39	1.15 (0.86-1.53)		.35	1.10 (0.80-1.52)		.57
	>4 h	1.43 (1.04-1.96)	.03	1.45 (1.05-2.01)		.03	1.53 (1.06-2.22)		.02

^a^Simple model.

^b^Adjusted for gender, age, and body mass index.

^c^Adjusted for gender, age, body mass index, demographics, and lifestyle factors.

^d^OR: odds ratio.

## Discussion

### Principal Findings

Mobile phone and TV were the most popular electronic media devices in our study population, but the usage was polarized by the age groups. Almost all the youth and young adults had mobile phones, while nearly two-thirds of the older adults did not use a mobile phone. On the contrary, over 90% of the older population treated TV viewing as a pastime, while relatively fewer young people watched TV. Another typical finding was that, unlike most previous studies, we found a quadratic curvilinear association between sleep quality and electronic device usage time; this J-shape occurred among all the participants for TV viewing and computer usage and among the youths for mobile phone usage.

Our study found that the prevalence of TV viewing in Macau was approximately 10% lower than that reported in some other countries and regions, such as the United States, the United Kingdom, Japan, Mainland China, and Hong Kong [2]. About half of the participants used computers daily, which was consistent with that reported in other Chinese populations in Mainland China and Hong Kong [2]. Regarding mobile phone usage, our study population exhibited a slightly higher prevalence than that reported in other countries and regions [2]. In our study, the longest number of hours was spent daily on mobile phone use, followed by TV viewing and computer use. Internet access is the key for people choosing electronic media devices. A smartphone can facilitate access to the internet rapidly and conveniently, which might be the reason for most participants to spend more time on the mobile phones instead of the traditional TV [20]. The percentage of users and viewing time of mobile phones and computers decreased with increasing age, which was consistent with that reported in previous studies in other countries [6,21]. Although the older population spent lesser time on mobile phones and computers, an increasing trend in using these devices was reported in a Chinese population [7].

The sleep duration in our study population was similar to that reported in previous studies conducted in Macau, Hong Kong, and Mainland China [7,22,23], which was around 7.3-7.8 hours. Females had poorer sleep quality than males, and they reported that they took a long time to fall asleep and were more likely to experience sleep disturbances. These findings were similar to that reported in a local study conducted among adolescents in 2012 [24] and that reported in a study on older adults in 2016 [8]. We found that sleep latency increased with age, which is consistent with the findings from a large cross-cultural study [25] conducted in China, Ghana, India, Mexico, and Russia, as well as in many other studies [26-28]. A recent study conducted among 0.5 million Chinese individuals suggested that increased sleep problems were associated with the growing use of TV, internet, mobile phones, and social media in the nighttime, which results in reduced sleep duration and increased prevalence of insomnia [22].

Most previous studies suggested an inverse linear relationship between electronic device usage and sleep problems [1]. However, our study found a significant quadratic curvilinear association, which was a “J” shape pattern in all 3 types of electronic device usage. The higher PSQI global score increased towards the end of the longer and shorter durations of electronic device usage. This pattern also appeared in the logistic regression analysis for poor sleep quality, in which people with a moderate duration of usage had the lowest odds to have a poor sleep status. Several mechanisms can be highlighted as the reasons for longer electronic device usage leading to poorer sleep quality. The electronic devices might affect sleep quality directly through reduced sleep duration and persistent electromagnetic radiation [29]. Bright light exposure from the electronic devices might suppress the release of melatonin and disrupt the circadian rhythm [10,29,30]. Long-time use of electronic devices may lead to physical discomforts such as muscular pain and headache, which could result in sleep problems [31]. All these reasons could explain how sleep quality was affected by long-time usage of screen-based electronic devices. Our study also found that moderate use of screen-based electronic devices resulted in the best sleep quality; the J-shape pattern indicating the mild use of these devices was better than that of nonusers or of those who used these devices for very short durations. A study conducted in the United Kingdom suggested that a moderate amount of digital-screen engagement such as watching TV, using a computer, or using a smartphone could help maintain the mental well-being and increase mood regulation and relaxation, which could improve sleep quality [32]. These screen-based electronic devices are widely used in modern society for recreation and relaxation. A study in Belgium showed that 36.7% of the adolescents considered TV viewing as a sleep aid for relaxation [33]. Another study in a Chinese population identified that watching TV and surfing the internet could improve happiness [34]. Some studies indicated that moderate use of smartphones could be considered as a coping strategy that diverts attention from stressful situations, which helps to regulate the individuals’ negative emotions [35,36]. Negative emotions and stressful situations can increase sleep fragmentation and decrease sleep duration, thereby resulting in a poor sleep status [37]. Based on our findings and the evidence from these previous studies, we suggest that moderate and appropriate use of screen-based electronic devices could be helpful in emotion regulation and stress relief, which enhances sleep quality thereafter.

### Limitations of Our Study

Our study has several limitations. First, the measurement of the electronic device usage did not specify the times, places, and contents watched. Moreover, the usage of other portable or screen-based devices such as video game machines were not evaluated in our study. Further, we did not measure the details of the smartphone usage pattern, for example, the time spent on making traditional phone calls and that spent on using apps based on interactive operations. Nor did we measure the details about when the phone calls were made (day or night), the frequency of the phone calls, and the content of the phone calls. Such information would have allowed for a more comprehensive and insightful investigation into the relationship between electronic device usage and sleep quality. Second, underestimation or overestimation might occur from self-reporting. The participants could tend to report a shorter or longer screening time for social desirability, which might affect the strength of the associations. However, we believe that this would not overturn the nature of the associations that we found because the biases in the responses were random. The large sample size would alleviate the effects of the self-reporting bias on the results.

### Future Research and Conclusions

Our study was the first to conduct a comprehensive investigation of the usage of 3 typically used screen-based electronic media devices, namely, TV, computer, and mobile phones and its relationships with sleep quality in a representative Chinese population with a wide range of ages. Most young people use mobile phones frequently in their daily lives, indicating that digital life is an integral part of the modern society. The most interesting finding in our study is that a typical “J” shape association was observed between sleep quality and TV viewing, computer usage, and mobile phone usage. The extreme use of screen-based electronic devices predicted poor sleep status, whereas moderate use was acceptable. These findings can be used to raise awareness among the general public on how to maintain the healthy use of screen-based electronic devices. As a public health issue, making age-specific guidelines for screen-based electronic device usage with regard to quantity and timing should be developed [29]. Future experimental and observational studies that elucidate how the pattern (frequency, duration, contents, location, time of the day, etc) of electronic device usage alters sleep and circadian rhythms across the life course and leads to poor health outcomes are needed. These studies could provide evidence for tailor-made primary prevention strategies in the future.

## Appendix

Multimedia Appendix 1 Average daily hours of the electronic media device usage, the PSQI global score, and poor sleep quality among the participants according to demographics and lifestyle factors.

Multimedia Appendix 2 Media device usage of the participants (N=1500).

Multimedia Appendix 3 Seven component scores of sleep quality by gender and age groups.

## Abbreviations

ANOVA: analysis of variance
BMI: body mass index
HLLL: healthy life, longer lives
OR: odds ratio
PSQI: Pittsburgh Sleep Quality Index
TV: television
WHO: World Health Organization
