# Supplementary material for: Relationships Between the Usage of Televisions, Computers, and Mobile Phones and the Quality of Sleep in a Chinese Population: Community-Based Cross-Sectional Study
Source: J Med Internet Res. 2020 Jul 7;22(7):e18095. doi: 10.2196/18095 (PMC7380995; doi:10.2196/18095)
Supplement: Multimedia Appendix 1 [file jmir_v22i7e18095_app1.doc]

Multimedia Appendix 1

Supplementary Table 1. Average daily hours of the electronic media device usage, the PSQI global score, and poor sleep quality among the participants according to demographics and lifestyle factors

|  | TV Mean (SD) | *P* ^c^ | Computer Mean (SD) | *P* ^c^ | Mobile Mean (SD) | *P* ^c^ |  | N | PSQI score, mean (SD) | *P* ^c^ | Poor sleep quality  N (%) | *P* ^c^ |
| --- | --- | --- | --- | --- | --- | --- | --- | --- | --- | --- | --- | --- |
| **Gender** |  | .03 |  | .04 |  | .02 |  |  |  | <.001 |  | <.001 |
| Male | 1.7 (1.5) |  | 1.7 (2.3) |  | 2.7 (2.4) |  |  | 641 | 4.49 (2.60) |  | 181 (28.2) |  |
| Female | 1.8 (1.8) |  | 1.4 (2.2) |  | 3.0 (2.5) |  |  | 783 | 5.04 (2.93) |  | 304 (38.8) |  |
| **Age Group** | < .001 | | < .001 | | < .001 | |  |  |  | .55 |  | .58 |
| Youth (15–24) | 1.1 (1.4) |  | 2.7 (2.6) |  | 4.4 (2.9) |  |  | 204 | 4.99 (2.67) |  | 71 (34.8) |  |
| Young Adult (25–44) | 1.3 (1.3) |  | 2.1 (2.5) |  | 3.3 (2.4) |  |  | 506 | 4.77 (2.80) |  | 174 (34.4) |  |
| Middle Age (45–64) | 2.0 (1.6) |  | 1.0 (1.8) |  | 2.4 (2.0) |  |  | 531 | 4.82 (2.89) |  | 186 (35.0) |  |
| Elderly (≥ 65) | 2.9 (1.6) |  | 0.2 (0.7) |  | 1.1 (1.8) |  |  | 183 | 4.58 (2.64) |  | 54 (29.5) |  |
| **Marital Status** | < .001 | | < .001 | | < .001 | |  |  |  | .22 |  | .60 |
| Single/Divorced/Separated | 1.4 (1.5) |  | 2.3 (2.7) |  | 3.5 (2.7) |  |  | 456 | 4.92 (2.84) |  | 160 (35.1) |  |
| Married/with partner | 2.0 (1.6) |  | 1.0 (1.8) |  | 2.4 (2.1) |  |  | 847 | 4.72 (2.78) |  | 285 (33.6) |  |
| **Educational Level** | < .001 | | < .001 | | < .001 | |  |  |  | .07 |  | .27 |
| Primary or Lower | 2.7 (1.6) |  | 0.5 (1.4) |  | 1.6 (2.0) |  |  | 263 | 4.65 (3.08) |  | 85 (32.3) |  |
| Secondary | 1.7 (1.6) |  | 1.4 (2.0) |  | 3.0 (2.4) |  |  | 726 | 4.96 (2.82) |  | 261 (36.0) |  |
| Tertiary or Higher | 1.2 (1.2) |  | 2.5 (2.7) |  | 3.3 (2.5) |  |  | 424 | 4.59 (2.57) |  | 134 (31.6) |  |
| **Employment Status** | < .001 | | < .001 | | < .001 | |  |  |  | .06 |  | .06 |
| Employed | 1.5 (1.4) |  | 1.7 (2.4) |  | 3.0 (2.4) |  |  | 981 | 4.86 (2.81) |  | 344 (35.1) |  |
| Retired | 3.1 (1.5) |  | 0.2 (0.7) |  | 1.0 (1.7) |  |  | 156 | 4.22 (2.68) |  | 39 (25.0) |  |
| Student | 1.0 (1.2) |  | 2.4 (2.5) |  | 4.0 (2.6) |  |  | 137 | 4.84 (2.80) |  | 45 (32.8) |  |
| Others (housewife, unable to work) | 2.4 (1.9) |  | 1.0 (1.9) |  | 2.5 (2.4) |  |  | 150 | 4.79 (2.80) |  | 57 (38.0) |  |
| **Monthly Household Income (MOP)** | < .001 | |  | .001 |  | .12 |  |  |  | .002 |  | <.001 |
| ≤ $9999 | 2.7 (2.1) |  | 1.0 (1.9) |  | 2.4 (2.7) |  |  | 95 | 5.39 (2.90) |  | 45 (47.4) |  |
| $10000 – $29999 | 1.6 (1.5) |  | 1.4 (2.1) |  | 2.9 (2.4) |  |  | 597 | 4.90 (2.89) |  | 219 (36.7) |  |
| $30000 – $59999 | 1.8 (1.5) |  | 1.5 (2.1) |  | 2.7 (2.2) |  |  | 398 | 4.59 (2.65) |  | 116 (29.1) |  |
| ≥ $60000 | 1.4 (1.2) |  | 1.9 (2.5) |  | 2.9 (2.6) |  |  | 112 | 4.07 (2.41) |  | 24 (21.4) |  |

(to be continued)

(Table 2 Continued)

|  | TV Mean (SD) | *P* ^c^ | Computer Mean (SD) | *P* ^c^ | Mobile Mean (SD) | *P* ^c^ |  | N | PSQI score, mean (SD) | *P* ^c^ | Poor sleep quality  N (%) | | | *P* ^c^ |  |
| --- | --- | --- | --- | --- | --- | --- | --- | --- | --- | --- | --- | --- | --- | --- | --- |
| **BMI** | .04 | |  | .008 |  | < .001 |  |  |  | <.001 |  | | | .009 |  |
| Underweight (≤ 18.5) | 1.7 (1.7) |  | 1.9 (2.3) |  | 3.5 (2.7) |  |  | 125 | 5.22 (2.88) |  | 48 (38.4) | | |  |  |
| Normal (18.6–22.9) | 1.7 (1.5) |  | 1.6 (2.3) |  | 3.0 (2.4) |  |  | 742 | 4.55 (2.66) |  | 224 (30.2) | | |  |  |
| Overweight (23–24.9) | 1.8 (1.6) |  | 1.4 (2.2) |  | 2.6 (2.3) |  |  | 280 | 4.69 (2.85) |  | 101 (36.1) | | |  |  |
| Obesity (≥ 25) | 2.0 (1.8) |  | 1.2 (2.2) |  | 2.4 (2.4) |  |  | 277 | 5.36 (2.96) |  | 112 (40.4) | | |  |  |
| **Enough Physical Activity ^a^** |  | .01 |  | .14 |  | .15 |  |  |  | .70 |  | | | .97 |  |
| Yes | 1.6 (1.5) |  | 1.6 (2.2) |  | 2.7 (2.2) |  |  | 425 | 4.76 (2.74) |  | 137 (32.2) | | |  |  |
| No | 1.8 (1.6) |  | 1.5 (2.3) |  | 2.9 (2.6) |  |  | 714 | 4.69 (2.80) |  | 231 (32.4) | | |  |  |
| **Unhealthy eating habits ^b^** | < .001 | |  | .001 | < .001 | |  |  | < .001 | | |  | <.001 | | |
| Yes | 1.9 (1.6) |  | 1.3 (2.2) |  | 2.6 (2.5) |  |  | 905 | 5.33 (2.85) |  | 373 (41.2) | |  | |  |
| No | 1.5 (1.6) |  | 1.7 (2.3) |  | 3.1 (2.4) |  |  | 515 | 3.85 (2.41) |  | 110 (21.4) | | |  |  |
| **Alcohol Drinking** |  | .003 |  | .495 |  | .73 |  |  |  | .86 |  | | | .72 |  |
| Yes | 1.5 (1.4) |  | 1.6 (2.4) |  | 2.8 (2.4) |  |  | 363 | 4.77 (2.59) |  | 121 (33.3) | | |  |  |
| No | 1.8 (1.6) |  | 1.5 (2.2) |  | 2.8 (2.5) |  |  | 1056 | 4.80 (2.87) |  | 363 (34.4) | | |  |  |
| **Smoking** |  | .68 |  | .005 |  | .88 |  |  |  | .02 |  | | | .08 |  |
| Yes | 1.7 (1.5) |  | 1.0 (2.2) |  | 2.8 (2.6) |  |  | 116 | 5.39 (2.68) |  | 48 (41.4) | | |  |  |
| No | 1.8 (1.6) |  | 1.6 (2.3) |  | 2.8 (2.4) |  |  | 1303 | 4.73 (2.79) |  | 433 (33.2) | | |  |  |

^a^ According to WHO (2018), sufficient physical activity means, throughout the week, doing at least 150 minutes of moderate–intensity aerobic physical activity, or at least 75 minutes of vigorous–intensity aerobic physical activity.

^b^ Inappropriate eating habits include hunger (e.g. delaying or skipping meal), dieting (e.g. weight losing, fasting), or overeating.

^c^ *P*–value was calculated by Independent sample t–test, Chi–Square test, and One–way ANOVA, where appropriate.
