# Supplementary material for: Relationships Between the Usage of Televisions, Computers, and Mobile Phones and the Quality of Sleep in a Chinese Population: Community-Based Cross-Sectional Study
Source: J Med Internet Res. 2020 Jul 7;22(7):e18095. doi: 10.2196/18095 (PMC7380995; doi:10.2196/18095)
Supplement: Multimedia Appendix 2 [file jmir_v22i7e18095_app2.doc]

Multimedia Appendix 2

Supplementary Table 2. Media Device Usage of the Participants. (N=1500)

|  | TV users | *p*^c^ | Computer users | *p*^c^ | Mobile users | *p*^c^ |
| --- | --- | --- | --- | --- | --- | --- |
| **Gender, n (%)** |  | .78 |  | .09 |  | .24 |
| Male | 528 (78.0) |  | 364 (54.0) |  | 568 (84.3) |  |
| Female | 648 (78.6) |  | 405 (49.6) |  | 708 (86.4) |  |
| **Age Group, n (%)** |  | < .001 |  | < .001 |  | < .001 |
| Youth (15–24) | 141 (66.8) |  | 179 (84.8) |  | 209 (99.1) |  |
| Young Adult (25–44) | 372 (70.3) |  | 357 (67.6) |  | 514 (97.2) |  |
| Middle Age (45–64) | 475 (85.3) |  | 214 (38.4) |  | 482 (86.4) |  |
| Elderly (≥ 65) | 182 (92.9) |  | 19 (9.7) |  | 71 (36.4) |  |
| Length of stay, mean (SD) | 36.4 (16.0) | < .001 | 30.4 (12.9) | < .001 | 32.5 (14.3) | < .001 |
| **Marital Status, n (%)** |  | < .001 |  | < .001 |  | < .001 |
| Single | 416 (71.1) |  | 412 (70.8) |  | 530 (90.6) |  |
| Married/with partner | 741 (82.8) |  | 350 (39.1) |  | 736 (82.2) |  |
| **Educational Level, n (%)** |  | < .001 |  | < .001 |  | < .001 |
| Primary or Lower | 267 (94.3) |  | 42 (14.9) |  | 154 (54.4) |  |
| Secondary | 580 (76.8) |  | 380 (50.3) |  | 683 (90.5) |  |
| Tertiary or Higher | 313 (70.5) |  | 344 (77.7) |  | 431 (97.1) |  |
| **Employment Status, n (%)** |  | < .001 |  | < .001 |  | < .001 |
| Employed | 772 (75.3) |  | 577 (56.4) |  | 950 (92.7) |  |
| Retired | 169 (98.3) |  | 14 (8.1) |  | 68 (39.5) |  |
| Student | 93 (66.0) |  | 119 (84.4) |  | 140 (99.3) |  |
| Others (housewife, unable to work, unemployed) | 112 (87.5) |  | 45 (35.2) |  | 97 (75.8) |  |
| **Monthly Household Income, n (%)** |  | .002 |  | < .001 |  | < .001 |
| ≤ $9000 | 88 (80.7) |  | 34 (31.5) |  | 70 (64.8) |  |
| $10000 – $29999 | 459 (73.9) |  | 297 (47.9) |  | 533 (85.8) |  |
| $30000 – $59999 | 346 (84.4) |  | 235 (57.2) |  | 374 (91.0) |  |
| ≥ $60000 | 89 (77.4) |  | 75 (65.8) |  | 100 (87.0) |  |
| Unknown | 177 (78.7) |  | 123 (54.7) |  | 189 (84.0) |  |
| **BMI** |  | .03 |  | < .001 |  | < .001 |
| Underweight (≤ 18.5) | 106 (76.8) |  | 88 (63.8) |  | 127 (92.0) |  |

(To be continued)

Supplementary Table (continued)

|  | TV users | *p*^c^ | Computer users | *p*^c^ | Mobile users | *p*^c^ |
| --- | --- | --- | --- | --- | --- | --- |
| Normal (18.6–22.9) | 586 (75.9) |  | 423 (54.8) |  | 680 (88.0) |  |
| Overweight (23–24.9) | 233 (80.1) |  | 132 (45.5) |  | 231 (79.7) |  |
| Obese (≥ 25) | 223 (84.2) |  | 115 (43.6) |  | 216 (81.5) |  |
| **Enough Physical Activity ^a^, n (%)** |  | .09 |  | < .001 |  | < .001 |
| Yes | 494 (80.3) |  | 366 (59.4) |  | 555 (90.1) |  |
| No | 650 (76.7) |  | 388 (45.9) |  | 694 (81.9) |  |
| **Inappropriate Eating Habits ^b^, n** (%) |  | .003 |  | < .001 |  | < .001 |
| Yes | 666 (81.2) |  | 363 (44.3) |  | 649 (79.2) |  |
| No | 495 (74.8) |  | 400 (60.5) |  | 619 (93.4) |  |
| **Drinking Habits, n (%)** |  | .92 |  | .40 |  | .046 |
| Yes | 289 (78.1) |  | 198 (53.5) |  | 328 (88.6) |  |
| No | 876 (78.4) |  | 569 (51.0) |  | 944 (84.4) |  |
| **Smoking, n (%)** |  | .61 |  | < .001 |  | .90 |
| Yes | 92 (76.7) |  | 38 (31.7) |  | 102 (85.0) |  |
| No | 1075 (78.6) |  | 728 (53.3) |  | 1168 (85.4) |  |
| ^a^ According to WHO (2018), sufficient physical activity means doing at least 150 minutes of moderate–intensity aerobic physical activity throughout the week or at least 75 minutes of vigorous–intensity aerobic physical activity.  ^b^ Inappropriate eating habits include hunger (e.g. delaying or skipping meal), dieting (e.g. weight losing, fasting), or overeating.  ^c^ *p*–value was calculated using the Independent sample t–test, Chi–square test, and One–way ANOVA, where appropriate. | | | | | | |
