# Supplementary material for: Relationships Between the Usage of Televisions, Computers, and Mobile Phones and the Quality of Sleep in a Chinese Population: Community-Based Cross-Sectional Study
Source: J Med Internet Res. 2020 Jul 7;22(7):e18095. doi: 10.2196/18095 (PMC7380995; doi:10.2196/18095)
Supplement: Multimedia Appendix 3 [file jmir_v22i7e18095_app3.doc]

Multimedia Appendix 3

Supplementary Table 3. Seven component scores of sleep quality by gender and age groups

|  | Gender | | |  | Age group | | | | |
| --- | --- | --- | --- | --- | --- | --- | --- | --- | --- |
|  | Male | Female | *P*–value |  | Youth  (15–24) | Young adult  (25–44) | Middle age  (45–64) | Elderly  (≥ 65) | *P*–value |
| Sleep duration (hours) | 7.26 (1.21) | 7.21 (1.41) | .48 |  | 7.28 (1.26) | 7.20 (1.26) | 7.23 (1.36) | 7.28 (1.42) | .84 |
| PSQI subjective sleep quality | 0.91 (0.66) | 1.01 (0.71) | .007 |  | 1.04 (0.75) | 0.95 (0.68) | 0.95 (0.69) | 0.97 (0.62) | .36 |
| PSQI sleep latency | 0.71 (0.78) | 0.90 (0.84) | <.001 |  | 0.94 (0.85) | 0.81 (0.79) | 0.81 (0.82) | 0.71 (0.80) | .04 |
| PSQI sleep duration | 0.76 (0.84) | 0.81 (0.92) | .32 |  | 0.81 (0.84) | 0.80 (0.86) | 0.79 (0.91) | 0.73 (0.92) | .79 |
| PSQI habitual sleep efficiency | 0.45 (0.81) | 0.53 (0.91) | .08 |  | 0.43 (0.79) | 0.54 (0.92) | 0.50 (0.86) | 0.45 (0.80) | .37 |
| PSQI sleep disturbances | 0.89 (0.57) | 0.97 (0.55) | .01 |  | 0.89 (0.50) | 0.88 (0.55) | 0.98 (0.59) | 1.00 (0.54) | .004 |
| PSQI use of sleeping medication | 0.08 (0.37) | 0.06 (0.32) | .24 |  | 0.04 (0.24) | 0.07 (0.32) | 0.09 (0.39) | 0.07 (0.35) | .31 |
| PSQI daytime dysfunction | 0.71 (0.60) | 0.74 (0.61) | .22 |  | 0.84 (0.60) | 0.72 (0.63) | 0.71 (0.61) | 0.65 (0.53) | .01 |
